# Temporal patterns of pre- and post-natal target organ damage associated with hypertensive pregnancy: a systematic review

**DOI:** 10.1093/eurjpc/zwad275

**Published:** 2023-08-22

**Authors:** Hannah Rebecca Cutler, Logan Barr, Prenali Dwisthi Sattwika, Annabelle Frost, Mohanad Alkhodari, Jamie Kitt, Winok Lapidaire, Adam James Lewandowski, Paul Leeson

**Affiliations:** Cardiovascular Clinical Research Facility, Division of Cardiovascular Medicine, Radcliffe Department of Medicine, University of Oxford, Headley Way, Headington, Oxford OX3 9DU, UK; Department of Biomedical and Molecular Sciences, Queens University, Barrie St, Kingston, Canada; Cardiovascular Clinical Research Facility, Division of Cardiovascular Medicine, Radcliffe Department of Medicine, University of Oxford, Headley Way, Headington, Oxford OX3 9DU, UK; Department of Internal Medicine, Faculty of Medicine, Public Health, and Nursing, Universitas Gadjah Mada, Bulaksumur, Caturtunggal, Kec, Kabupaten Sleman, Indonesia; Cardiovascular Clinical Research Facility, Division of Cardiovascular Medicine, Radcliffe Department of Medicine, University of Oxford, Headley Way, Headington, Oxford OX3 9DU, UK; Cardiovascular Clinical Research Facility, Division of Cardiovascular Medicine, Radcliffe Department of Medicine, University of Oxford, Headley Way, Headington, Oxford OX3 9DU, UK; Healthcare Engineering Innovation Center, Department of Biomedical Engineering, Khalifa University, Abu Dhabi, Shakhbout Bin Sultan St, Hadbat Al Za'faranah, United Arab Emirates; Cardiovascular Clinical Research Facility, Division of Cardiovascular Medicine, Radcliffe Department of Medicine, University of Oxford, Headley Way, Headington, Oxford OX3 9DU, UK; Cardiovascular Clinical Research Facility, Division of Cardiovascular Medicine, Radcliffe Department of Medicine, University of Oxford, Headley Way, Headington, Oxford OX3 9DU, UK; Cardiovascular Clinical Research Facility, Division of Cardiovascular Medicine, Radcliffe Department of Medicine, University of Oxford, Headley Way, Headington, Oxford OX3 9DU, UK; Cardiovascular Clinical Research Facility, Division of Cardiovascular Medicine, Radcliffe Department of Medicine, University of Oxford, Headley Way, Headington, Oxford OX3 9DU, UK

**Keywords:** Hypertension, Pregnancy, Trajectories, Phenotype, Complications

## Abstract

**Aims:**

Hypertensive pregnancy is associated with increased risks of developing a range of vascular disorders in later life. Understanding when hypertensive target organ damage first emerges could guide optimal timing of preventive interventions. This review identifies evidence of hypertensive target organ damage across cardiac, vascular, cerebral, and renal systems at different time points from pregnancy to postpartum.

**Methods and results:**

Systematic review of Ovid/MEDLINE, EMBASE, and ClinicalTrials.gov up to and including February 2023 including review of reference lists. Identified articles underwent evaluation via a synthesis without meta-analysis using a vote-counting approach based on direction of effect, regardless of statistical significance. Risk of bias was assessed for each outcome domain, and only higher quality studies were used for final analysis. From 7644 articles, 76 studies, including data from 1 742 698 pregnancies, were identified of high quality that reported either blood pressure trajectories or target organ damage during or after a hypertensive pregnancy. Left ventricular hypertrophy, white matter lesions, proteinuria, and retinal microvasculature changes were first evident in women during a hypertensive pregnancy. Cardiac, cerebral, and retinal changes were also reported in studies performed during the early and late post-partum period despite reduction in blood pressure early postpartum. Cognitive dysfunction was first reported late postpartum.

**Conclusion:**

The majority of target organ damage reported during a hypertensive pregnancy remains evident throughout the early and late post-partum period despite variation in blood pressure. Early peri-partum strategies may be required to prevent or reverse target organ damage in women who have had a hypertensive pregnancy.

**See the editorial comment for this article ‘Assessment of target organ damage in hypertensive pregnancy’, by E. The, https://doi.org/10.1093/eurjpc/zwad319.**

## Introduction

Pregnancy is characterised by extensive cardiac, vascular, renal, and neurological changes to accommodate the increased haemodynamic demands of the foetus, whilst ensuring the health of the mother.^1,2^ When the maternal cardiovascular system is unable to adjust to these demands, such as due to poor placentation or pre-existing cardiac dysfunction, adaptations occur to maintain placental perfusion. These adaptations underlie the rise in blood pressure pathognomonic for pregnancy hypertension.

The increased peripheral resistance, adverse left ventricular remodelling and altered renal and cerebral blood flow^1^ are similar to the adverse adaptations described at other times in life as hypertensive target organ damage.^3^ This may explain why women who have hypertensive pregnancies are up to four times more likely to have later vascular diseases such as hypertension, myocardial infarctions and strokes.^4–6^ However, recent trials indicate that this association can be disrupted by changing how women are cared for postpartum. Interventions during the first six weeks after delivery can result in lower blood pressure for up to three years.^4,7,8^ These sustained benefits are most likely explained by beneficial remodelling of the cardiac and vascular target organ damage that occurs during a hypertensive pregnancy.^9^

To understand the breadth of potential benefits from early preventive interventions, we performed a systematic review of observational studies to identify evidence for changes across the brain, vasculature, heart, and renal systems during a hypertensive pregnancy, which could be considered hypertensive target organ damage. We then reviewed to what extent these continue to be reported as evident in the immediate, early, and later post-partum periods. Finally, whether these patterns of target organ changes parallel patterns of blood pressure variation or follow distinct trajectories.

To our knowledge, no other reviews have compared the temporal patterns of target organ damage across the whole body during and after hypertensive disorders of pregnancy. By understanding the timepoints at which target organ damage occurs, we can identify windows at which trialled interventions can be targeted to reverse damage and improve future clinical outcomes.

## Methods

This systematic review was conducted in accordance with the Synthesis Without Meta-analysis (SWiM)^10^ reporting guidelines.

### Search strategy and selection criteria

The search strategy was developed by combining key terms and medical subject headings related to target phenotype organ damage and women with hypertensive disorders of pregnancy (*Table 1*). A systematic search was performed in Ovid/MEDLINE and EMBASE to identify scientific reports in the English language published until February 2022. Restrictions for English language, human studies, and full text availability were used. Records identified through database searches were imported to Rayann software for deduplication and study selection by two independent reviewers (H.R.C. and L.B.).

**Table 1:** Search terms and combinations used for PubMed and Embase searches

Sources	Search terms
PubMed and Embase	(((preeclampsia OR pre-eclampsia OR eclampsia) OR ((hypertension hypertensive) AND (pregnancy OR gestational))) OR (‘Hypertension, Pregnancy-Induced’)) AND (((phenotype* OR imaging OR modelling OR remodelling OR ‘machine learning’ OR ‘disease progression*’ OR ‘disease exacerbation*’ OR life course*) OR (modelling OR remodelling)) OR (‘Multimodal Imaging”[Mesh])).

Titles, abstracts, and full texts identified by the searches were reviewed against the eligibility criteria as detailed in *Table 2*. Studies obviously irrelevant determined based on title and abstract were excluded and duplicates removed. Abstracts fulfilling inclusion criteria were selected for full-text review. Abstracts with selection discrepancies were discussed, and a decision was made based on these discussions. If no consensus, was reached, then a third reviewer made the final decision (P.D.S.). Additional searches were conducted by one reviewer (H.R.C.) in ClinicalTrials.gov and Google (last search February 2023). Reference lists of retrieved reports and relevant reviews were manually searched for potentially eligible titles as well.

**Table 2:** Selection criteria for study eligibility assessment

	Inclusion criteria	Exclusion criteria
Exposure	Hypertensive disorders of pregnancy including superimposed hypertension, gestational hypertension, pre-eclampsia, pregnancy-induced hypertension, eclampsia and haemolysis, elevated liver enzymes, and low platelets syndrome	Primary focus on other maternal conditions e.g. gestational diabetes
Outcome	Hypertensive target organ damage and temporal changes in blood pressure	—
Time frame	Any time during or after pregnancy	Time frame is not stated
Study design	Original research study, human studies, trend studies, cohort studies, panel designs, and time-series designs	Abstracts, oral communications, guides, guidelines, reviews, systematic reviews, meta-analyses, case reports, opinion papers, and posters of congress
Availability	Able to access full-text portable document format	Non-English language.

Inclusion criteria included primary research studies and longitudinal follow-up studies including trend studies, cohort studies, panel designs, and time-series designs published since the year 2000. Exclusion criteria were abstracts, oral communications, guides, guidelines, reviews, and posters of congresses. A focus was on papers reporting mothers who have had a hypertensive disorder of pregnancy including pre-eclampsia, eclampsia, gestational hypertension, superimposed hypertension and haemolysis, elevated liver enzymes, and low platelets syndrome that reported on target organ damage. They must have either stated the timepoints at which the measures were taken or that the mothers had a ‘history’ of hypertension during pregnancy. Measures must have been taken during pregnancy or postpartum.

Studies which examined temporal changes in blood pressure during pregnancy and postpartum were also included, but they were not classed as target organ damage. The reason for this was to understand the phenotype trajectory of blood pressure during hypertensive pregnancy so that the time course of target organ damage could be compared with the times at which blood pressure alterations occur. Studies with mixed populations were included only if outcome data were stratified by patient population.

### Outcomes

The outcome of this review was target organ damage related to hypertensive disorders of pregnancy occurring during pregnancy or postpartum. Target organ damage was defined as negative alterations affecting the structure and function of organ systems in the body, this was based on clinical thresholds from academic literature. We did not classify risk of disease or death as target organ damage (e.g. cardiovascular disease risk). As such, studies which only examined disease risk were not included in the synthesis. Nonetheless, risk of disease and morbidity was discussed.

The primary outcome was cardiac changes (i.e. remodelling, hypertrophy, left and right atrioventricular morphology indices, diastolic and systolic dysfunction, or fibrosis). Secondary outcomes were vascular changes (i.e. narrowing, widening, thickening, or stiffening of blood vessels, remodelling, calcification, or changes in vessel or microvascular function), renal changes (i.e. serum creatinine, proteinuria, glomerular filtration rate, and structural alterations), and neural changes (i.e. vasogenic oedema, white matter lesions, blood brain barrier leakage, changes in grey matter volume or total brain volume, as well as cognitive disturbances). We also examined temporal changes in blood pressure that occurred during pregnancy or postpartum.

### Data extraction

All outcomes listed in the included studies were extracted using a standardized extraction form by two reviewers (H.R.C. and L.B.). Each reviewer extracted the data and then checked over each other’s collected information for accuracy. Any disagreement was resolved by discussion until consensus was reached and if consensus was not reached, then a third reviewer made the final decision (P.D.S.). General study information was extracted including first author, year of publication, country, study design, study period, inclusion and exclusion criteria, total number of women with hypertensive, normotensive or uncomplicated pregnancies, outcomes per group and overall conclusions, strengths, and limitations.

For each outcome, the following information was collected when available: measurement tools, investigators, measured and reported timepoints, unit of measures, effect sizes and corresponding *P*-values, as well as any reported subgroup analysis. When a specific study had findings published in multiple articles, unique data were extracted, and these reports were treated as a single study. Studies with mixed populations had outcome data extracted only when pertaining to those with hypertensive disorders of pregnancy. Additionally, for studies with multiple measures, primary measures of the study were included.

### Risk-of-bias assessment

A risk of bias of selected studies was assessed independently by two authors (P.D.S. and H.R.C.) using the Newcastle–Ottawa scale.^11^ The Newcastle–Ottawa scale assigns a maximum of nine points for the least risk of bias in three domains: (i) selection of study groups (four points); (ii) comparability of groups (two points); and (iii) ascertainment of exposure and outcomes (three points) for case–control and cohort studies. Studies that scored zero to three points were classed as high risk of bias, four to six points were classed as moderate risk and seven to nine points were classed as low risk. Any discrepancies were addressed by re-evaluation of the original article to reach consensus. Due to the heterogeneity of the data, a meta-analysis was not conducted.

### Data synthesis

Findings were organized according to outcome domains (cardiac, vascular, renal, neural, and blood pressure). They were then ordered according to the study timeframe with the maximum length of follow-up used for ordering. Risk of bias was tabulated alongside the outcomes. Due to the heterogeneity of the outcomes, no attempts were made to transform results into a standardized metric. For each study, effect sizes were summarized as reported by the authors of included studies. Evidence was then synthesized using a vote-counting approach based solely on direction of effect. When available data permitted, direction of effect was defined for each outcome domain and type of effect size measure was labelled as ‘non-favourable outcome’ or ‘no difference’.

A ‘non-favourable outcome’ was reported if hypertensive disorders of pregnancy were linked with worsening target organ damage outcomes. If >70% of outcomes reported similar direction between the normotensive and hypertensive pregnancy populations, then studies were labelled as ‘no difference’. Direction of effect was summarized based on the difference in changes between groups or if no comparison, direction of effect was based on whether there was any evidence of target organ damage. We explored sources of heterogeneity qualitatively by comparing study designs, exposures, disease outcome, and statistical methods. Findings from each individual study were graphically presented using the Harvest plot. Studies with final sample size in the experimental arm of <25 were defined as small, 25–99 as medium, and >100 as large. Conclusions were drawn based on the directness in relation to the review question.

## Results

**Figure 1:**
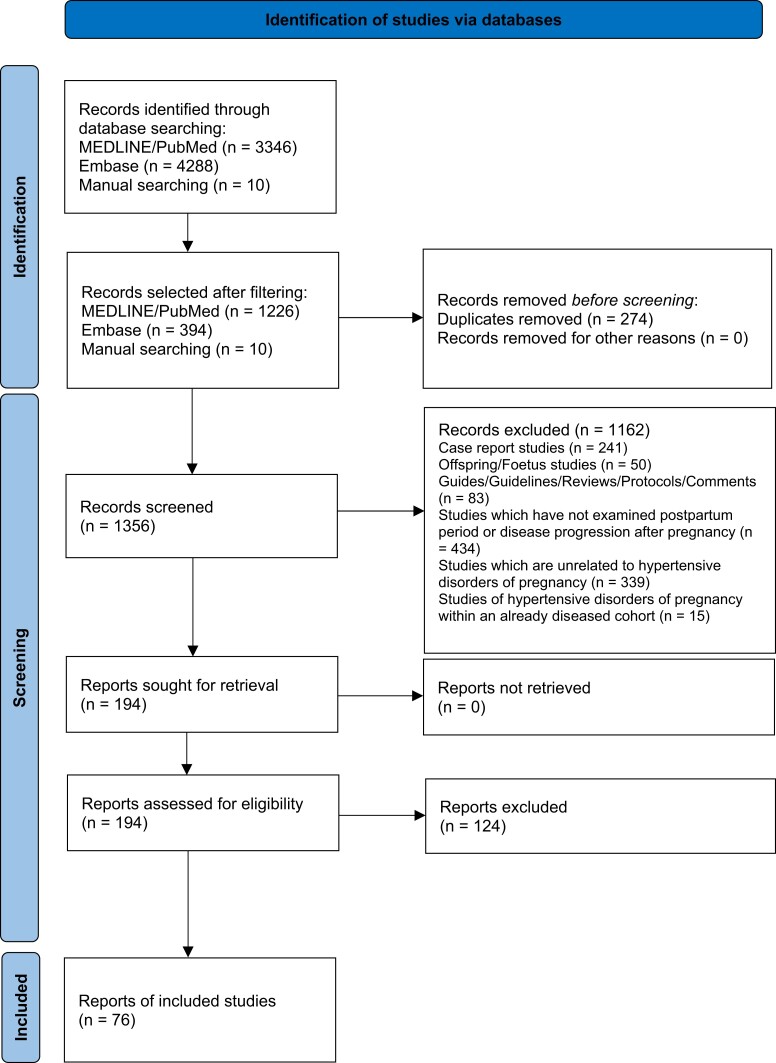
PRISMA flowchart of study selection process.

A total of 7644 records were identified in databases, registries, and other sources (*Figure 1*). After applying filters fitting the eligibility criteria, 1630 papers were selected. A further 274 duplicate papers were removed, leaving 1356 articles to be screened. After title and abstracts were screened, 1162 papers were removed, leaving 194 articles which were read in full. Of these, 76 were accepted as eligible for inclusion.

### Study characteristics

*Table 3* displays detailed characteristics of studies stratified by either blood pressure or type of target organ damage and the time at which the population were studied. A total of 76 studies including 15 observational (19.7%), 39 cohort (51.3%), 21 case–control (27.6%), and 1 randomized controlled trial (1.31%) were selected with a collective total of 1 742 698 pregnancies. Pre-eclampsia was the predominant hypertensive disorder examined across the studies. However, the studies also examined women with eclampsia, gestational hypertension, pregnancy-induced hypertension and haemolysis-elevated liver enzymes, and low platelets syndrome.

**Table 3:** Characteristics of studies included in review

Authors	Study design	Population	Sample size (*N*)	Length of follow-up (mean ± SD, median ± IQR)	Outcome	Main results	Risk of bias
Giorgione *et al*.^12^	Prospective observational	Total pregnancies	*N* = *30*	4.5 (2–8) days before and 3.5 (2–6) days after birth.	Cardiac	LV hypertrophy/remodelling was detected in 21 patients. No significant difference in cardiac morphology indices such as LVMI (78.9 ± 16.3 vs. 77.9 ± 15.4 g/m^2^; *P* = 0.611) or RWT (0.45 ± 0.1 vs. 0.44 ± 0.1; *P* = 0.453) before vs. after LV delivery. Diastolic function did not demonstrate any peri-partum variation, with similar LA volume (52.4 ± 15.3 vs. 51.0 ± 15.6 mL; *P* = 0.433), lateral *E*′ (0.12 ± 0.03 vs. 0.12 ± 0.03 m/s; *P* = 0.307) and *E*/*E*′ ratio (7.9 ± 2.2 vs. 7.9 ± 1.7; *P* = 0.934) before vs. after delivery. Systolic function indices, such as LVEF (57.5 ± 3.4 vs. 56.4 ± 2.1%; *P* = 0.295) and GLS (−15.3 ± 2.6% vs. −15.1 ± 3.1%; *P* = 0.582), unchanged before vs. after delivery	*****
		PE	*N* = 20				Moderate
		GH	*N* = *10*				5
Castleman *et al*^13^	Cohort	Total pregnancies	*N* = 75	13 ± 1 weeks of gestation	Cardiac	SBP in HDP group at 13 ± 1 weeks gestation was higher than both other groups (*P* < 0.001 vs. Group 2; *P* = 0.007 vs. Group 3). Women with previous HDP had increased A wave and increased AI. Women without prior HTN had more compliance to controls. This adaptation was not in pregnancy with prior HTN, where increased arterial stiffness was observed	*******
		HDP	*N* = 25				Moderate
		Normotensive	*N* = 50				7
		Non-pregnant	*N* = 40				
Vaught *et al*.^14^	Cohort	Total pregnancies	*N* = 99	33.1 ± 3.6 weeks gestation 31.8 ± 4.9 weeks gestation	Cardiac	PEC had higher RVSP (31.0 ± 7.9 vs. 22.5 ± 6.1 mmHg; *P* < 0.001) and decreased GRLVSS (−19.6 ± 3.2% vs. −23.8 ± 2.9% *P* < 0.0001) to controls. For left-sided cardiac parameters, differences (*P* < 0.001) in mitral septal *e*′ velocity (9.6 ± 2.4 vs. 11.6 ± 1.9 cm/s), septal *E*/*e*′ ratio (10.8 ± 2.8 vs. 7.4 ± 1.6), LA area size (20.1 ± 3.8 vs. 17.3 ± 2.9 cm^2^), and posterior and septal wall thickness [median (IQR): 1.0 cm (0.9–1.1 cm) vs. 0.8 cm (0.7–0.9 cm), and 1.0 cm (0.8–1.2 cm) vs. 0.8 cm (0.7–0.9 cm)]	********
		PEC	*N* = 63				
		Controls	*N* = 36				Low
							8
Hieda *et al*.^15^	Case–control	Total pregnancies	*N* = 41	4–8 weeks gestation, 32–36 weeks gestation and 6–10 weeks pp	Cardiac	Pre-pregnancy, no differences in BP and HR between groups. Corrected isovolumic relaxation time was longer, *E*/*e*′ was larger, and Tei index was greater in the high-risk group than controls. Rate of HDP during the study was significantly greater in high-risk group than controls [OR = 8.94 (95% CI: 1.55–51.5), *P* = 0.007]. Pre-pregnancy *E*/*e*′ was an independent predictor for HDP (*P* = 0.017)	*****
		GH	*N* = 16				Moderate
		Controls	*N* = 25				5
Foo *et al*.^16^	Prospective observational	Total pregnancies	*N* = 530	Preconception to 6–10 weeks pp	Cardiac	PE group had lower preconception CO (4.9 vs. 5.8 L/min; *P* = 0.002) and CI (2.9 vs. 3.3 L/min per m^2^; *P* = 0.031) while MAP (87.1 vs. 82.3 mmHg; *P* = 0.05) and TPR (1396.4 vs. 1156.1 dynes s/cm^5^; *P* < 0.001) were higher. Longitudinal trajectories for CO and TPR were similar between PE and healthy pregnancies, but PE showed a more exaggerated fall in MAP in first trimester, followed by a steeper rise and fall to pp values	******
		PE	*N* = 15				Moderate
							6
Tyldum *et al*.^17^	Cohort	Total pregnancies	*N* = 40	35 weeks gestation and 3 months pp	Cardiac	Diastolic function reduced in PE, with lower ‘*e*’, and higher ratio of ‘*E*/*e*’. Early diastolic mitral inflow deceleration time and isovolumetric relaxation time were similar between the groups. ‘S’ was lower in PE group in pregnancy. Both diastolic and systolic LV functions normalized pp. FMD impaired in PE group during pregnancy and three months pp	*****
		PE	*N* = 20				Moderate
		Controls	*N* = 20				5
Yu *et al*.^18^	Cohort	Total pregnancies	*N* = 82	33–36 weeks gestation to 3 months pp	Cardiac	GH group had significantly lower mean LV peak GLS to controls. PE patients had significantly lower GL, circumferential, and radial strain compared with controls	******
		GH	*N* = 27				Moderate
		PE	*N* = 25				6
		Controls	*N* = 30				
Valensise *et al*.^19^	Cohort	Total pregnancies	*N* = 1345	20 weeks gestation to term and 1 year pp.	Cardiac	5 of 32 patients with late PE and 45 of 75 patients with early PE had bilateral notching of the uterine artery at 24 weeks (15.6 vs. 60.0%; *P* < 0.05). TVR was 1605 ± 248 vs. 739 ± 244 dyns/cm^5^, and CO was 4.49 ± 1.09 vs. 8.96 ± 1.83 L in early vs. late PE (*P* < 0.001). Pre-pregnancy BMI was higher in late vs. early PE (28 ± 6 vs. 24 ± 2 kg/m^2^; *P* < 0.001)	*******
		LO PE	*N* = 32				Low
		EO PE	*N* = 75				7
		Controls	*N* = 1238				
Hamad *et al*.^20^	Cohort	Total pregnancies	*N* = 65	3–6 months pp	Cardiac	Significant differences in LV and LA dimensions and function between groups. Higher septal and lateral *E*/*E*′ ratio (*P* < 0.0001, 0.0008). *E*/*E*′ ratio lateral levels higher in EO PE necessitating delivery before 34 weeks gestation than those who got PE and delivered ≥ 34 weeks (*P* = 0.0004, 0.005)	****
		PE	*N* = 35				Moderate
		Controls	*N* = 30				4
Kalapotharakos *et al*.^21^	Cohort	Total pregnancies	*N* = 14	6 months pp	Cardiac	LV mass at 1–3 days pp was higher after severe LO-PE (57 g/m^2^) to controls (48 g/m^2^; *P* = 0.01). PWV decreased between 1 and 3 days and 6 months pp after PE (6.1–5.0 m/s; *P* = 0.028). No difference in PWV 1–3 days pp after PEC compared after normal pregnancy (6.1 vs. 5.6 m/s; *P* = 0.175). BP normalized within 6 months in all but 1 patient	*****
		Severe PE	*N* = 6				Moderate
		Controls	*N* = 8				5
Ghossein-doha *et al*.^22^	Cohort	Total pregnancies	*N* = 349	9 months pp (6–19)	Cardiac	Formerly PE women had increased LVMi and decreased cardiac diastolic function at post-partum screening	*******
		HDP	*N* = 27				Moderate
		Controls	*N* = 322				6
Valensise *et al*.^23^	Case–control	Total pregnancies	*N* = 222	12–18 months pp	Cardiac	22/75 patients developed recurrent PE. In non-pregnant state, patients with recurrent PE to controls and non-recurrent PE had lower SV (63 ± 14 vs. 73 ± 12 and 70 ± 11 mL, *P* < 0.05), CO (4.6 ± 1.2 vs. 5.3 ± 0.9 and 5.2 ± 1.0 L, *P* < 0.05), higher *E*/*E*′ ratio (11.02 ± 3.43 vs. 7.34 ± 2.11 vs. 9.03 ± 3.43, *P* < 0.05), and higher TVR [1638 ± 261 dynes(−1)cm(−5) vs. 1341 ± 270 dynes(−1)cm(−5) and 1383 ± 261 dynes(−1)cm(−5), *P* < 0.05]. LVMI higher in recurrent and non-recurrent PE compared with controls [30.0 ± 6.3 g/m(2.7) and 30.4 ± 6.8 g/m(2.7) vs. 24.8 ± 5.0 g/m(2.7), *P* < 0.05]	*******
		Prior PE	*N* = 75				Low
		Controls	*N* = 147				7
Melchiorre *et al*.^24^	Case–control study	Total pregnancies	*N* = 142	1–2 years pp	Cardiac	1 year pp LV dysfunction/hypertrophy significantly higher in preterm PE (56%) compared with term PE (14%) or matched controls (8%; *P* values <0.001)	*****
		PE	*N* = 64				Moderate
		Controls	*N* = 78				5
Breetveld *et al*.^25^	Observational	Total pregnancies	*N* = 100	0.8 and 4.8 years pp	Cardiac	Low PV associated with concentric remodelling (OR 4.37; 95% CI, 1.06–17.40; and adjusted OR, 4.67; 95% CI, 1.02–21.42). SBP, DBP, and MAP also associated with concentric remodelling [OR, 1.15 (95% CI, 0.99–1.35); OR, 1.24 (95% CI, 0.98–1.58); and OR, 1.20 (95% CI, 0.98–1.47), respectively]	****
		PE	*N* = 100				Moderate
							4
Vaught *et al*.^26^	Observational	Total pregnancies	*N* = 33	33–36 weeks gestation and 4 years pp	Cardiac	PE had thicker LV posterior walls on initial antenatal echocardiogram compared with 52% who did not develop HTN [1.0 cm (0.9–1.1 cm) vs. 0.9 cm (0.7–0.9 cm). *P* < 0.016]	******
		Severe PE	*N* = 33				Moderate
							6
Orabona *et al*.^27^	Case–control	Total pregnancies	*N* = 90	4 months-6 years pp	Cardiac	Significant LV fibrosis in EO-PE to LO-PE and controls (*P* < 0.001 for CIBSIVS and *P* = 0.005 for cIBSPW), whereas aortic fibrosis did not differ significantly	******
		EO-PE	*N* = 30	2.3 ± 0.7 years			Moderate
		LO-PE	*N* = 30	2.5 ± 0.8 years			6
		Controls	*N* = 30	2.2 ± 0.6 years			
Orabona *et al*.^28^	Case–control	Total pregnancies	*N* = 169	4 months−6 years	Cardiac	HELLP showed significant LV concentric hypertrophy (20.4%). HELLP and PE groups, LV concentric remodelling (46.9 and 46.7%, respectively), diastolic dysfunction and reduced LVEF (14.3 and 21.7%, respectively) were documented. RV variables did not differ significantly, except for FAC and *E*/*E*′ ratio being slightly impaired in HELLP to PE (16.3 vs. 10.0%, *P* = 0.04; 14.3 vs. 3.3%, *P* = 0.03, respectively)	******
		PE	*N* = 60	2.3 ± 0.8 years			Moderate
		HELLP	*N* = 49	2.8 ± 1.1 years			6
		Control	*N* = 60	2.2 ± 0.6 years			
Bergen *et al*.^29^	Case–control	Total pregnancies	*N* = 4912	6 years pp (5.7–7.2)	Cardiac	PE and GH had six-fold increased risk to develop CH after pregnancy to controls (OR 6.6, 95% CI 4.6–9.5). Women with PE ha higher BP and higher risk of CH (OR 4.5, 95% CI 2.6–7.8) at follow-up to controls also	******
		GH	*N* = 205				Moderate
		PE	*N* = 95				6
		Controls	*N* = 4612				
Ghossein-Doha *et al*.^30^	Cross-sectional cohort	Total pregnancies	*N* = 148	4–10 years pp	Cardiac	Asymptomatic HF-B prevalence was ∼3.5-fold higher in PE group to controls (25 vs. 7%, *P* < 0.01); 67% of this group had concentric remodelling and 22% had mildly impaired EF. After adjustment, PE significantly associated with HF-B [adjusted OR, 4.4 (95% CI, 1.0–19.1]	*****
		PE	*N* = 107	4.8(4.0–6.3) years			Moderate
		Controls	*N* = 41	7.8 (6.3–9.9)			5
Boardman *et al*.^31^	Case–control	Total pregnancies	*N* = 173	5–10 years pp	Cardiac	HDP had distinct cardiac geometry with higher LVMI (49.9 ± 7.1 vs. 46.0 ± 6.5 g/m^2^; *P* = 0.001) and EF (65.6 ± 5.4 vs. 63.7 ± 4.3%; *P* = 0.03) but lower GLS (−18.31 ± 4.46 vs. −19.94 ± 3.59%; *P* = 0.02). LAVI was also increased (40.4 ± 9.2 vs. 37.3 ± 7.3 mL/m^2^; *P* = 0.03) and *E*:*A* reduced (1.34 ± 0.35 vs. 1.52 ± 0.45; *P* = 0.003). Aortic compliance (0.240 ± 0.053 vs. 0.258 ± 0.063; *P* = 0.046) and functional capillary density (105.4 ± 23.0 vs. 115.2 ± 20.9 capillaries/mm^2^; *P* = 0.01) reduced	******
		HDP	*N* = 103				Moderate
		Controls	*N* = 70				6
Ersbøll *et al*.^32^	Cohort	Total pregnancies	*N* = 84	7–10 years pp	Cardiac	Mean LVEF in PE was 69% and in uncomplicated pregnancies was 67%, respectively (*P* < 0.0001)	******
		PPCM	*N* = 28	7.58 years			Moderate
		PE	*N* = 28	7.92 years			6
		Controls	*N* = 28	8.42 years			
Demartelly *et al*.^33^	Cohort	Total pregnancies	*N* = 46	10 years pp	Cardiac	PE had worse GLS (−18.3 vs. −21.3%, *P* = 0.001), LV posterior wall thickness (0.91 vs. 0.80 mm, *P* = 0.003), and interventricular septal thickness (0.96 vs. 0.81 mm, *P* = 0.0002)	*******
		PE	*N* = 21				Low
		Controls	*N* = 25				7
Ghossein-Doha *et al*.^22^	Case–control	Total pregnancies	*N* = 28	1 and 14 years pp	Cardiac	LV geometry and systolic function comparable in groups at both time points. Age-related decline in *E*/*A* ratio and increase in intraventricular septum thickness noted in both groups over time, without appreciable differences between groups	******
		PE	*N* = 20				Moderate
		Controls	*N* = 8				6
Countouris *et al*.^34^	Case–control	Total pregnancies	*N* = 132	10 −11 years pp	Cardiac	HDP history had higher interventricular septal thickness (β = 0.08; *P* = 0.04) and RWT (β = 0.04; *P* = 0.04). In subgroup analyses, both HDP history and current hypertension had a higher LV remodelling (79.0%) to other groups [only HDP (36.4%; *P* = 0.01), only current HTN (46.2%; *P* = 0.02), and neither HDP nor HTN (38.2%; *P* < 0.001)], and lower mitral inflow *E*/*A* and annular *e*′	*******
		PE	*N* = 21	9.5 ± 0.9 years			Low
		GH	*N* = 9	9.8 ± 0.7 years			7
		Controls	*N* = 102				
Quesada *et al*.^35^	Case–control	Total pregnancies	*N* = 346	History of HDP	Cardiac	HDP history associated with 3.2-fold increased odds of HTN. Women with a history of HDP and HTN had higher ` measured LV mass compared with women with HDP only (99.4 ± 2.6 vs. 87.7 ± 3.2 g, *P* = 0.02). Similar frequency of LGE scar and trend towards increased LGE scar size (5.1 ± 3.4 vs. 8.0 ± 3.4 g, *P* = 0.09) among HDP history to women without	****
		HDP	*N* = 68				Moderate
		Controls	*N* = 278				4
Scantlebury *et al*.^36^	Cohort	Total pregnancies	*N* = 2637	History of HDP	Cardiac	HDP had a greater risk of LVH (OR: 1.42, 95% CI 1.01–1.99, *P* = 0.05). When duration of HTN was accounted, this relationship was no longer significant (OR: 1.19, CI 0.08–1.78 *P* = 0.38). Women with HDP also had greater LA size and lower mitral *E*/*A* ratio after adjusting for demographic variables. The prevalence of systolic dysfunction was similar between groups	******
		HDP	*N* = 427				Moderate
		Normotensive	*N* = 2210				6
Chandran *et al*.^37^	Observational	Total pregnancies	*N* = 150	26–39 weeks gestation	Vascular	Ocular symptoms in 22% of severe PE and in 100% of eclampsia patients. Fundus changes were seen in 48.7% of subjects. Arteriolar narrowing was most common finding	****
		Severe PE	*N* = 141				Moderate
		Eclampsia	*N* = 9				4
Rasdi *et al*.^38^	Prospective observational	Total pregnancies	*N* = 154	35 weeks gestation – 6 weeks pp	Vascular	32.5% had hypertensive retinopathy. 98% had visual acuity 6/6, while 100% gained 6/6 pp. Generalized arteriolar narrowing was most common retinopathy	****
		HDP	*N* = 154				Moderate
							4
Brueckmann *et al*.^39^	Cohort	Total pregnancies	*N* = 601	1st, 2nd and 3rd trimester and 19.1 ± 15.3 weeks pp	Vascular	AVR in PE was lower (*P* < 0,02) in T1, T2, and pp compared with normotensives (T1: 0.80 ± 0.06 vs. 0.9 ± 0.08; T2: 0.86 ± 0.06 vs. 0.9 ± 0.11; T3: 0.88 ± 0.09 vs. 0.89 ± 0.1; pp: 0.83 ± 0.08 vs. 0.87 ± 0.1)	***
		GH	*N* = 38				High
		PE	*N* = 143				3
		Controls	*N* = 420				
		Non-pregnant	*N* = 50				
Herman *et al*.^40^	Cohort	Total pregnancies	*N* = 38	<20 weeks gestation, 20 weeks-delivery and 2–6 months pp	Vascular	Macular thicknesses diminished at <20 weeks gestation in pregnancies with HDP (mean 3.94 μm; 95% CI 4.66, 3.21) and 11 controls (mean 3.92 μm; 5.05, 2.79; *P* < 0.001 vs. non-pregnant dimensions in both; *P* = 0.983 HDP vs. controls	*****
		HDP	*N* = 27				Moderate
		Controls	*N* = 11				5
Hamad *et al*.^41^	Cohort	Total pregnancies	*N* = 65	3–6 months pp	Vascular	FMD was decreased in PE at inclusion and at follow-up (*P* < 0.05). FMD was lower in EO PE to LO PE (*P* = 0.018, 0.002 and 0.039)	****
		PE	*N* = 35				Moderate
		Controls	*N* = 30				4
Yuan *et al*.^42^	Case–control	Total pregnancies	*N* = 63	34 weeks gestation to 16–20 months pp	Vascular	LV and carotid arterial remodelling more frequent in PE than controls (96 vs. 40%, 82 vs. 48%, both *P* < 0.0001). Carotid CSA greater in PE vs. controls (11.23 ± 0.17 vs. 8.58 ± 1.88 mm^2^, *P* < 0.00001). Carotid stiffness and IMT correlated with cardiac diastolic function and BP (*P* < 0.05). Both *E_a_* and *E_es_* greater in PE, vs. controls (*E_a_*: 2.41 ± 0.57, vs. 1.98 ± 0.46 mmHg/mL, *P* = 0.0005; *E_es_*: 11.68 ± 9.51 vs. 6.91 ± 6.13 m/s^2^,*P* = 0.002). Carotid remodelling persisted 16–20 months pp in PE and NT	******
		PE	*N* = 23				Moderate
		Controls	*N* = 40				6
Akhter *et al*.^43^	Cohort	Total pregnancies	*N* = 119	27–37 weeks gestation and 1 year pp	Vascular	Thicker mean common carotid artery intima, thinner media, and higher I/M ratio in PE to NT (mean I/M difference, 0.21; 95% CI, 0.17–0.25; *P* < 0.0001). After adjustment for T1 BMI and MAP, differences in thickness and I/M remained significant. About 1-year pp, these values improved but group differences remained significant (all adjusted *P* < 0.0001)	*******
		PE	*N* = 55				Low
		Controls	*N* = 64				7
Soma-Pillay *et al*.^44^	Case–control	Total pregnancies	*N* = 80	1 year pp	Vascular	Corrected central retinal arteriolar equivalent and corrected central retinal venular equivalent were lower in PE compared with controls both at delivery and 1 year pp (*P* < 0.001)	*******
		Severe PE	*N* = 40				Low
		Controls	*N* = 40				7
Sciatti *et al*.^45^	Case–control	Total pregnancies	*N* = 169	2.3 ± 0.8 years pp	Vascular	HELLP associated with larger aortas than PE or controls. Aortic elastic properties, including *E_a_*, similar between HELLP and PE. *E_es_* more impaired in HELLP. 25% of women who experienced HELLP had a VAC, whereas only 5% of PE did	******
		PE	*N* = 60	2.8 ± 1.1 years			Moderate
		HELLP	*N* = 49	2.2 ± 0.6 years			6
		Controls	*N* = 60				
Breetveld *et al*.^46^	Cohort	Total pregnancies	*N* = 104	> 4 years pp	Vascular	FMD lower in PE vs. controls (6.12 vs. 8.22%, *P* < 0.01); history of PE remained associated independently with lower FMD after adjusting for metabolic syndrome risk factors and obstetric parameters (β, −1.88; 95% CI, −3.59 to −0.18)	*****
		PE	*N* = 67	5.3 (4.4–6.4)			Moderate
		Controls	*N* = 37	8.3 (6.6–9.9)			5
Barr *et al*.^47^	Case–control	Total pregnancies	*N* = 60	6 months – 5 years pp	Vascular	Severe PE had significantly higher vasodilation to acetylcholine and sodium nitroprusside to controls (*P* < 0.01; *P* = 0.03) and prior mild PE (*P* = 0.03; *P* < 0.01). Neither degree of post occlusive reactive hyperaemia (*P* = 0.98), nor time to return halfway to baseline [OR = 1.026 (0.612, 1.72); *P* = 0.92], differed between PE and controls	*******
				104.0 ± 80.7 weeks			Low
		Severe PE	*N* = 16	76.3 ± 55.8 weeks			7
		Mild PE	*N* = 14	121.1 ± 69.2 weeks			
		Controls	*N* = 30				
Barr *et al*.^48^	Case–control	Total pregnancies	*N* = 60	6 months – 5 years	Vascular	No differences in CIMT between PE and controls. Left CIMT significantly thicker than right CIMT in PE but not controls. Carotid artery diameter did not vary between PE and controls. Right side far wall circumferential strain did not differ between women with PE history and normotensive pregnancy. Left carotid artery far wall circumferential strain was lower in PE to controls. Severe PE had greatest decrease in carotid artery far wall circumferential strain	*******
							Low
		PE	*N* = 30	91.1 ± 70.5 weeks			7
		Controls	*N* = 30	121.1 ± 69.2 weeks			
Benschop *et al*.^49^	Case–control	Total pregnancies	*N* = 3391	6.2 years	Vascular	PE group had smaller retinal arteriolar calibres 6 years pp than controls (adjusted difference: −0.40 SDS; 95% CI:−0.62,−0.19). For women with previous GH, similar trends were observed (−0.20 SDS; 95% CI:−0.34,−0.05)	*******
		PE	*N* = 63	range 5.7–7.4			Low
		GH	*N* = 145				7
		Controls	*N* = 3183				
Akhter *et al*.^50^	Cohort	Total pregnancies	*N* = 86	11 ± 5 years	Vascular	History of severe PE had thicker CCA intima and higher I/M vs. NT, also after adjustment for MAP, BMI and CCA intima–media thickness (IMT) (all *P* < 0.0001). CCA-IMT did not differ between groups. In receiver–operating characteristics curve analysis, intima thickness and I/M discriminated between those with and without previous PE [(AUC), 0.98 and 0.93], whereas CCA-IMT did not (AUC, 0.52)	******
		PEC	*N* = 42	11 ± 5 years			Moderate
		Normotensive	*N* = 44	11 ± 5 years			6
White *et al*.^51^	Cohort	Total pregnancies	*N* = 80	32–37 years	Vascular	CAC score frequency >50 Agatston units was greater in PE (23 vs. 0%, *P* = 0.001). Odds of a higher CAC score was 3.54 (CI, 1.39–9.02) and 2.61 (CI, 0.95–7.14) times greater in prior PE without and with adjustment for current hypertension, respectively. After adjustment for BMI, CAC odds based on PE history remained significant at 3.20 (CI, 1.21–8.49)	*****
		PE	*N* = 40	34.9 (32.9− 36.7)			Moderate
		Controls	*N* = 40	34.5 (33.6–36.7)			5
Kountouris *et al*.^52^	Cohort	Total pregnancies	*N* = 687	6–8 weeks pp	Renal	Renal dysfunction in 32% of cohort and in 46 and 22% of women with and without PE. Independent predictors were PE, CH, antenatal serum creatinine, highest 24-h urinary protein, and BP ≥ 140/90 mmHg at post-natal visit	*****
		HDP	*N* = 687				Moderate
							5
Kaze *et al*.^53^	Prospective cohort	Total pregnancies	*N* = 54	6 weeks, 3 months and 6 months pp	Renal	Significant improvement in BP, renal function, and proteinuria in follow-up (*P* < 0.002). 13 patients with renal failure recovered within 6 weeks. 26, 17 and 1 patients had persisting proteinuria at 6 weeks, 3 months and 6 months pp, respectively	*******
		PEC	*N* = 37				Low
		Eclampsia	*N* = 17				7
Ishaku *et al*.^54^	Prospective cohort	Total pregnancies	*N* = 488	9 weeks, 6 months, 1 year pp	Renal	HDPs more likely have decreased eGFR to controls (12, 5.7, 4.3% vs. 0, 2 and 2.4%, respectively). eGFR varied with HDP subtypes with PE/eclampsia showing higher prevalence than CH and GH	******
		HDP	*N* = 410				Moderate
		Controls	*N* = 78				6
Kaleta *et al*.^55^	Cohort	Total pregnancies	*N* = 83	12 months	Renal	Most PE had proteinuria persistence (>120 mg/L after delivery) 6 months (*P* = 0.02) and 12 months pp (*P* < 0.0001) to controls. Also reduced GFR persisted up to 6 months pp in PE patients to controls (*P* < 0.001)	*******
		PE	*N* = 44				Low
		Healthy controls	*N* = 39				7
Strevens *et al*.^56^	Prospective controlled cohort	Total pregnancies	*N* = 36	20 months pp	Renal	Glomerular endotheliosis present in all women with PE and GH, and in 5/12 controls, although significant differences in the degree of endotheliosis were found between the groups	****
		HDP	*N* = 36				Moderate
							4
Berks *et al*.^57^	Observational	Total pregnancies	*N* = 205	1.5, 3, 6−, 12−, 18− and 24-months post-delivery	Renal	At 3 months pp, 39% had HTN, decreased to 18% at 2 years pp. Resolution time increased by 60% (*P* < 0.001) for every 10 mmHg increase in max SBP and 40% (*P* = 0.044) for 10 mmHg increase in max DBP. At 3 months pp, 14% had proteinuria, decreased to 2% at 2 years pp. Resolution time increased by 16% (*P* = 0.001) for 1 g/day increase in proteinuria	*****
		PE	*N* = 205				Moderate
							5
Sandvik *et al*.^58^	Case–control	Total pregnancies	*N* = 158	10.9 ± 1.0 years pp	Renal	Urinary ACR 0.53 mg/mmol for PE and 0.50 mg/mmol for NT (*P* = 0.54). Only 1 PE had ACR >2.5 mg/mmol in two of three urine samples. PE not associated with urinary ACR above 75th percentile. PE not have higher eGFR than women without PE (107.9 vs. 104.9 mL/min per 1.73 m^2^; *P* = 0.12), but preterm PE associated with eGFR above the 75th percentile (*P* = 0.03)	********
		PE	*N* = 89				Low
		Controls	*N* = 69				8
Shammas *et al*.^59^	Case–control	Total pregnancies	*N* = 147	10 years pp	Renal	Women with PE and PIH had higher risk of HTN 10 years later to controls (23% for PE, and 39% for PIH vs. 3% for control). Albumin corrected calcium levels significantly higher in patients with PE (2.41 mmol/L) and PIH (2.42 mmol/L) vs. control (2.33 mmol/L) as well as sig. diff in microalbuminuria (23% in PE, and 16% in PIH vs. 3% in control)	*******
		PE	*N* = 47				Low
		PIH	*N* = 54				7
		Controls	*N* = 46				
Bergman *et al*.^60^	Cohort	Total pregnancies	*N* = 15	During pregnancy	Neural	Women with eclampsia had increased CSF concentrations of all pro-inflammatory cytokines and TNF-alpha compared with normotensive pregnancies and also for interleukin-6 and TNF-alpha compared with women with PE. Women with PE also showed increases in pro-inflammatory cytokines IL-6 and IL-8 but not TNF-alpha in the CSF to women with normotensive pregnancies. eclampsia and PE showed increase in CSF to plasma albumin ratio to normotensive women	*****
		PE	*N* = 4	27.3 weeks			Moderate
		Eclampsia	*N* = 4	18.3 weeks			4
		Controls	*N* = 7	30.9 weeks			
Singh *et al*. (2021)^61^	Observational	Total pregnancies	*N* = 30	0–7 days pp	Neural	Eight women had PRES features. The most specific parameters for predicting PRES were age (<24 years), platelet count (<0.69 lacs/mm^3^), serum ALT (>129 IU/L) and AST (>55 IU/L), total bilirubin (>1.3 mg/dL), low haemoglobin (<8.7 g/dL) and presence of seizures. The most sensitive predicting parameters were serum uric acid > 5.2 mg/dL, SBP > 164 mm Hg, DBP > 100 mmHg and serum creatinine > 0.8 mg/dL	***
		Eclampsia	*N* = 7				High
		PE	*N* = 11				3
		GH	*N* = 9				
		CH	*N* = 3				
Zeeman *et al*.^62^	Observational	Total pregnancies	*N* = 27	38.7 (± 2.4 weeks) and 6–8 weeks pp	Neural	All but 2/27 women (93%) had reversible vasogenic oedema. 6 also had areas of cytotoxic oedema consistent with cerebral infarction. 5/6 women had persistent imaging of infarction when studied pp, however, without clinical neurologic deficits	****
		Eclampsia	*N* = 27				Moderate
							4
Zeeman *et al*.^63^	Cohort	Total pregnancies	*N* = 21	36.8 ± 2.6 weeks (PE) and 37.2 ± 1 weeks (Control) and 6–8 weeks pp (Both)	Neural	T3 large cerebral artery blood flow significantly higher in PE. Mean vessel diameter was unchanged, except for the left posterior cerebral artery. No difference in vessel diameter or cerebral blood flow between the groups when not pregnant	*****
		PE	*N* = 12				Moderate
		Controls	*N* = 9				5
Soma-Pillay *et al*.^64^	Cohort	Total pregnancies	*N* = 94	6 months and 1 year	Neural	Cerebral WML’s in 61.7% of women at delivery, 56.4% at 6 months and 47.9% at 1 year. Majority of lesions in the frontal lobes. Lesions presence at 1 year pp was associated with no. of drugs to control BP during pregnancy (OR 5.1, 95% CI 2.3–11.3, *P* < 0.001). Prevalence of WMLs at 1 year double in women with chronic HTN at 1 year to NT women (65.1 vs. 32.3%)	*****
		Severe PE	*N* = 60				Moderate
		HELLP	*N* = 34				5
Aukes *et al*.^65^	Cohort	Total pregnancies	*N* = 136	5.1 ± 3.7 years pp	Neural	Women who have had eclampsia scored significantly higher on the Cognitive Failures Questionnaire, compared with healthy parous control subjects (43.5 ± 14.6 vs. 36.1 ± 13.9, respectively; *P* < 0.05)	*****
		PE	*N* = 31				Moderate
		Eclampsia	*N* = 30				5
		Controls	*N* = 75				
Canjels *et al*.^66^	Case–control	Total pregnancies	*N* = 72	5–8 years pp	Neural	PE women had higher local efficiency in the prefrontal cortex (*P* = 0.048) and anterior cingulate cortex (*P* = 0.03) but lower local efficiency and local clustering coefficient in the amygdala (*P* = 0.004 and *P* = 0.02, respectively) and parahippocampal cortex (*P* = 0.007 and *P* = 0.008, respectively)	******
		PE	*N* = 55	5.6 years			Moderate
		Controls	*N* = 17	8.0 years			6
Aukes *et al*.^67^	Cohort	Total pregnancies	*N* = 68	6.4 ± 5.6 years pp	Neural	Women with eclampsia demonstrated subcortical white matter lesions more than twice as often when compared with controls (41 vs. 17%; odds ratio, 3.3; 95% CI, 1.05–10.61; *P* = 0.04)	*******
		Eclampsia	*N* = 39				Low
		PE	*N* = 29				7
Wiegman *et al*.^68^	Cohort	Total pregnancies	*N* = 213	7.6 ± 4.7 years pp	Neural	Formerly PE and eclamptic women have WMLs more often (34.4% compared with 21.3%; *P* > 0.05) and more severely (0.07 compared with 0.02 mL; *P* > 0.05) than controls. In all women, most lesions was located in the frontal lobes followed by the parietal, insular, and temporal lobes	*******
		Eclampsia	*N* = 64	5.2 ± 4.1 years			Low
		PE	*N* = 74	5.0 ± 3.3 years			7
		Controls	*N* = 75				
**Canjels** *et al*.^69^	Cohort	Total pregnancies	*N* = 35	1–13 years pp	Neural	Leakage rate and fractional leakage volume higher in formerly PE women than controls in global white (*P* = 0.001) and grey (*P* = 0.02) matter. Regionally, the frontal (*P* = 0.04) and parietal (*P* = 0.009) cortical grey matter, and frontal (*P* = 0.001), temporal (*P* < 0.05) and occipital (*P* = 0.007) white matter showed higher leakage rates in formerly PE women. Odds of a high leakage rate after PE were generally higher in white-matter than grey-matter regions	*******
		PE	*N* = 22	6.6 ± 3.2 years			Low
		Controls	*N* = 13	9.0 ± 3.7 years			7
**Siepmann** *et al*.^70^	Case–control	Total pregnancies	*N* = 83	5–15 years pp	Neural	Previously PE women had reduced cortical grey matter volume (523.2 6 30.1 vs. 544.4 6 44.7 mL, *P*, 0.05) and WML changes were more extensive in previously PE women. They displayed increased temporal lobe white matter disease (lesion volume: 23.2 6 24.9 vs. 10.9 6 15.0 mL, *P*, 0.05) and altered microstructural integrity (radial diffusivity: 538 6 19 vs. 526 6 18 3 1026 mm^2^/s, *P*, 0.01), which also extended to occipital and parietal lobes	*******
		PE	*N* = 34	7.8 ± 1.7 years			Low
		Controls	*N* = 49	8.7 ± 2.3 years			7
Postma *et al*.^71,72^	Cohort	Total pregnancies	*N* = 145	7 years pp	Neural	Both PE and eclamptic women performed worse on motor functions domain (*P* < 0.05), without differences on the other domains. They scored worse on the Cognitive Failures Questionnaire (*P* < 0.01), the Hospital Anxiety and Depression Scale anxiety (*P* < 0.01), and depression (*P* < 0.05) subscales	*****
		Eclampsia	*N* = 46	8 (2–20)			Moderate
		PE	*N* = 51	6(1–18)			
		Controls	*N* = 48	6(1–27)			
Adank *et al*.^73^	Cohort	Total pregnancies	*N* = 596	15 years pp (median 14.7 years, 90% range [13.9–16.1]).	Neural	HDP negatively associated with the 15-word learning test: immediate recall [−0.25, 95% CI (−0.44 to −0.06)] and delayed recall [−0.30, 95% CI (−0.50 to −0.10)]. Women with GH perform significantly worse on their 15-word learning test than women with a previous normotensive pregnancy	******
		GH	*N* = 80				Moderate
		PE	*N* = 35				6
		Controls	*N* = 481				
Dayan *et al*.^74^	Cohort	Total pregnancies	*N* = 568	Median 18 years pp (range 1–25).	Neural	PE scored significantly lower on Digit Symbol Substitution Test than women with normotensive (73.21 ± 14.79 vs. 75.87 ± 15.22; *P* = 0.047) and on the 3rd trial of Stroop Test (correct answers: 38.85 ± 3.62 vs. 39.42 ± 1.87; *P* = 0.014; completion time: 44.02 ± 10.48 vs. 41.62 ± 10.61 s; *P* = 0.01), but no differences in Rey Auditory Verbal Learning Test	*****
		PE	*N* = 193				Moderate
		Controls	*N* = 375				5
**Miller** *et al*.^75^	Cohort	Total pregnancies	*N* = 64	30–40 years pp	Neural	Multivariable modelling of individual cognitive tests revealed a differential association for letter fluency by pregnancy history (test for interaction *P* = 0.023); this score correlated with the aortic haemodynamic index in the PE (partial *R*^2^ = 0.20), but not the NP (partial *R*^2^ = 0.00) group	*******
		PE	*N* = 34	35.4 ± 3.2 (34.9, 33.0–37.1)			Low7
		Controls	*N* = 30	35.4 ± 2.7 (34.5, 33.7–36.2)			
Fields *et al*.^76^	Cohort	Total pregnancies	*N* = 80	35–40 years pp	Neural	No statistically significant differences in raw scores on tests of cognition and mood between women with PE histories to NT. However, a consensus diagnosis of mild cognitive impairment or dementia trended towards greater frequency in women with histories of PE to those with NT (20 vs. 8%, *P* = 0.10) and affected more domains among the PE group (*P* = 0.03), most strongly related to executive dysfunction (*d* = 1.96) and verbal list learning impairment (*d* = 1.93)	*****
		PE	*N* = 40	34.9 years (32–47.2)			Moderate
		Control	*N* = 40	34.5 years (32–46.4)			5
Miller *et al*.^75^	Cohort	Total pregnancies	*N* = 64	35 ± 3 years	Neural	Aortic haemodynamics was associated with the cognitive index, whether considering a potential interaction with pregnancy history (*P* = 0.035) or not (*P* = 0.026) (interaction *P* = 0.178). There was also a differential association for letter fluency by pregnancy history (test for interaction *P* = 0.023); this score correlated with the aortic haemodynamic index in the PE (partial *R*^2^ = 0.20), but not the NP (partial *R*^2^ = 0.00) group	*****
		PE	*N* = 34				Moderate
		Control	*N* = 30				5
Mielke *et al*.^77^	Observational	Total pregnancies	*N* = 1279	History of HDP	Neural	HDP histories performed worse on all measures of processing speed [Digital Symbol Substitution Test (mean score, 41.2 vs. 43.4; *P* = 0.005), Trail Making Test Part A (mean seconds, 45.1 vs. 42.2; *P* = 0.035), and Stroop (mean score, 173.9 vs. 181.0; *P* = 0.002)] and had smaller brain volumes compared with women with histories of normotensive pregnancies (286 vs. 297; *P* = 0.023)	****
		HDP history	*N* = 1279				Moderate
							4
Gunderson *et al*.^78^	Observational	Total pregnancies	*N* = 174,925	0–20 weeks’ gestation	BP	Adjusted OR, 95% CI, for increasing, stable, and elevated-stable groups were 3.25 (2.7–3.9), 5.3 (4.5–6.3), and 9.2 (7.7–11.1) for PE/eclampsia, and 6.4 (4.9–8.3), 13.6 (10.5–17.7), and 30.2 (23.2–39.4) for GH compared with declining groups. Ethnicity, and obesity modified trajectory-group associations to PE/eclampsia (*P* < 0.01)	*******
							Low
		PE/eclampsia	*N* = 4322				7
		GH	*N* = 8342				
		Normotensives	*N* = 162,261				
Hauspurg *et al*.^79^	Observational	Total pregnancies	*N* = 8899	11.6 ± 1.5 weeks gestation and 19.0 ± 1.6 weeks gestation	BP	Elevated BP (RR 1.54 95% CI, 1.18–2.02 of HDP) and stage 1 HTN (RR 2.16, 95% CI, 1.31–3.57 of HDP), DBP and SBP trajectories significantly linked with HDP (*P <* 0.01)	*******
							Low
							7
Roell *et al*.^80^	Case–control	Total pregnancies	*N* = 3319	17–34 weeks’ gestation	BP	Five BP trajectories, steeper BP clusters were associated with worse pregnancy outcomes, regardless of starting BP	*******
		PE	*N* = 1906				Low
		Controls	*N* = 1413				7
Nobles *et al*.^81^	Randomized controlled trial	Total pregnancies	*N* = 568	Preconception-36 weeks’ gestation	BP	BP (RR 1.13, 95% CI 1.02–1.25 per 2 mmHg increase in MAP at 4 weeks gestation associated with later development of PE)	********
		PE	*N* = 23				Low
		GH	*N* = 27				8
		Controls	*N* = 518				
Mukhtarova *et al*.^82^	Observational	Total pregnancies	*N* = 897	Preconception to 42 days (6 weeks) pp	BP	Peak SBPs and DBPs were on pp days 3,4,5, and 5,6,7, respectively. SBP fell below preconception after Day 15; DBP reached plateau after Day 17 and was above preconception till Day 42 (*P* < 0.001). BP stabilization/resolution were days 11 (95% CI: 10–12) and 23 (95% CI: 21–25). By Day 42, 91 and 74.1% of women achieved BP stabilization and resolution	*****
							Moderate
							5
Benschop *et al*.^83^	Observational	Total pregnancies	*N* = 200	1 year	BP	1 year pp, 41.5% of women had hypertension with ABPM, Masked hypertension was most common (17.5%) followed by sustained hypertension (14.5%) and WCH (9.5%)	*****
		Severe PE	*N* = 200				Moderate
							5
Veerbeek *et al*.^84^	Observational	Total pregnancies	*N* = 748	<5 years	BP	Almost half of the EO-PE women had developed hypertension, as opposed to 39 and 25% of women in the pregnancy-induced hypertension and LO-PE groups, respectively	******
		EO-PE	*N* = 448				Moderate
		LO-PE	*N* = 76				6
		PIH	*N* = 224				
Heida *et al*.^85^	Cohort	Total pregnancies	*N* = 22,265	7.7 years	BP	Women with HDP reported hypertension 7.7 years earlier (95% CI 6.9–8.5) than controls. After confounder adjustment, HDP was associated with presence of hypertension at enrolment (OR 2.12, 95% CI 1.98–2.28) and onset of CVD later in life (HR 1.21, 95% CI 1.10–1.32)	*****
							Moderate
		HDP	*N* = 6157				5
		GDM	*N* = 1089				
		Controls	*N* = 15019				
Behrens *et al*.^7^	Cohort	Total pregnancies	*N* = 1,508,090	10 years	BP	Cumulative incidences of HTN in 20, 30, or 40 s in women whose 1st pregnancy with HDP were 13.7, 20.3, and 32.4% and in controls were 4.0, 5.7, and 11.3%)	********
							Low
		**HDP**	*N* = 23,235				8
		Controls	*N* = 1,484,855				
Bokslag *et al*.^86^	Cohort	Total pregnancies	*N* = 187	9 – 16 years	BP	SBP 126 ± 18.6 mmHg in EO PE and 115 ± 17.0 in uncomplicated pregnancy, *P* < 0.0001; DBP 82 ± 9.8 mmHg in EO PE and 74 ± 9.4 mmHg in uncomplicated pregnancy, *P* < 0.0001)	*********
		EO PE	*N* = 131	13.1 ± 2.2			Low
		Controls	*N* = 56	14.2 ± 2.3			9

HDP, hypertensive disorders of pregnancy; GH, gestational hypertension; CH, chronic hypertension; NT, normotensive pregnancy; PE, pre-eclampsia; PP, postpartum; *P*, probability; BP, blood pressure; OR, odds ratio; CI, confidence interval; DBP, diastolic blood pressure; SBP, systolic blood pressure; HTN, hypertension; MAP, mean arterial pressure; WCH, white coat hypertension; ABPM, ambulator blood pressure monitoring; *N*, number; EO-PE, early onset pre-eclampsia; LO-PE, late onset pre-eclampsia; CVD, cardiovascular disease; PIH, pregnancy-induced hypertension; PEC, pre-eclampsia with severe features; AVR, atrioventricular ratio; T1, first trimester; T2, second trimester; T3, third trimester; FMD, flow mediated dilation; LV, left ventricular; CSA, cross-sectional area; IMT, intima-media thickness; I/M, intima/media; HELLP, haemolysis, elevated liver enzyme, low platelets syndrome; VAC, vacuum-assisted biopsy; CIMT, carotid intima-media thickness; SDS, standard deviation score; CCA, coronary artery calcification; AUC, area under curve; LVMI, left ventricular mass index; RWT, relative wall thickness; GLS, global longitudinal strain; LA, left atrium; TPR, total peripheral resistance; CO, cardiac output; PWV, pulse wave velocity; CIBSIVS, calibrated integrated backscatter at the basal interventricular septum; HF-B, heart failure-B; EF, ejection fraction; WML, white matter lesions; eGFR, endothelial glomerular filtration rate; PRES, posterior reversible encephalopathy syndrome; ACR, urine albumin to creatinine ratio; ALT, alanine transaminase; AST, aspartate aminotransferase; BMI, body mass index; CAC, coronary artery calcification; CSF, cerebrospinal fluid; FAC, fractional area change; GDM, gestational diabetes mellitus; GRLVSS, global relative left ventricular systolic strain; HR, heart rate; IQR, interquartile range; LAVI, left atrial volume index; LGE, late gadolinium enhancement; LVEF, left ventricular ejection fraction; LVH, left ventricular hypertrophy; NP, natiuretic peptide; PPCM, postpartum cardiomyopathy; PV, pulse velocity; RR, relative ratio; RV, right ventricle; RVSP, right ventricular systolic pressure; SV, stroke volume; TNF, tumor necrosis factor; TVR, tricuspid regurgitation velocityt; cIBSIVS, calibrated integrated backscatter at the interventricular septum; CIBSPW, calibrated integrated backscatter at the posterior wall; CMRI, cardiomagnetic resonance imaging.

Altogether, 13 studies examined vascular changes (17.1%), 28 studies examined cardiac alterations (36.8%), 8 studies examined renal changes (10.5%), 17 studies examined neural changes (22.4%), and 10 studies examined blood pressure trajectories (13.1%). A total of 30 studies (39.4%) observed women during pregnancy and early postpartum (up to 1 year after pregnancy). Whilst 43 studies (56.5%) examined women late postpartum (one or more years postpartum). Additionally, three studies (3.9%) stated that the women had a ‘history’ of hypertensive pregnancy without defining the exact number of years postpartum. Study follow-ups ranged from preconception to up to 40 years postpartum.

**Figure 2:**
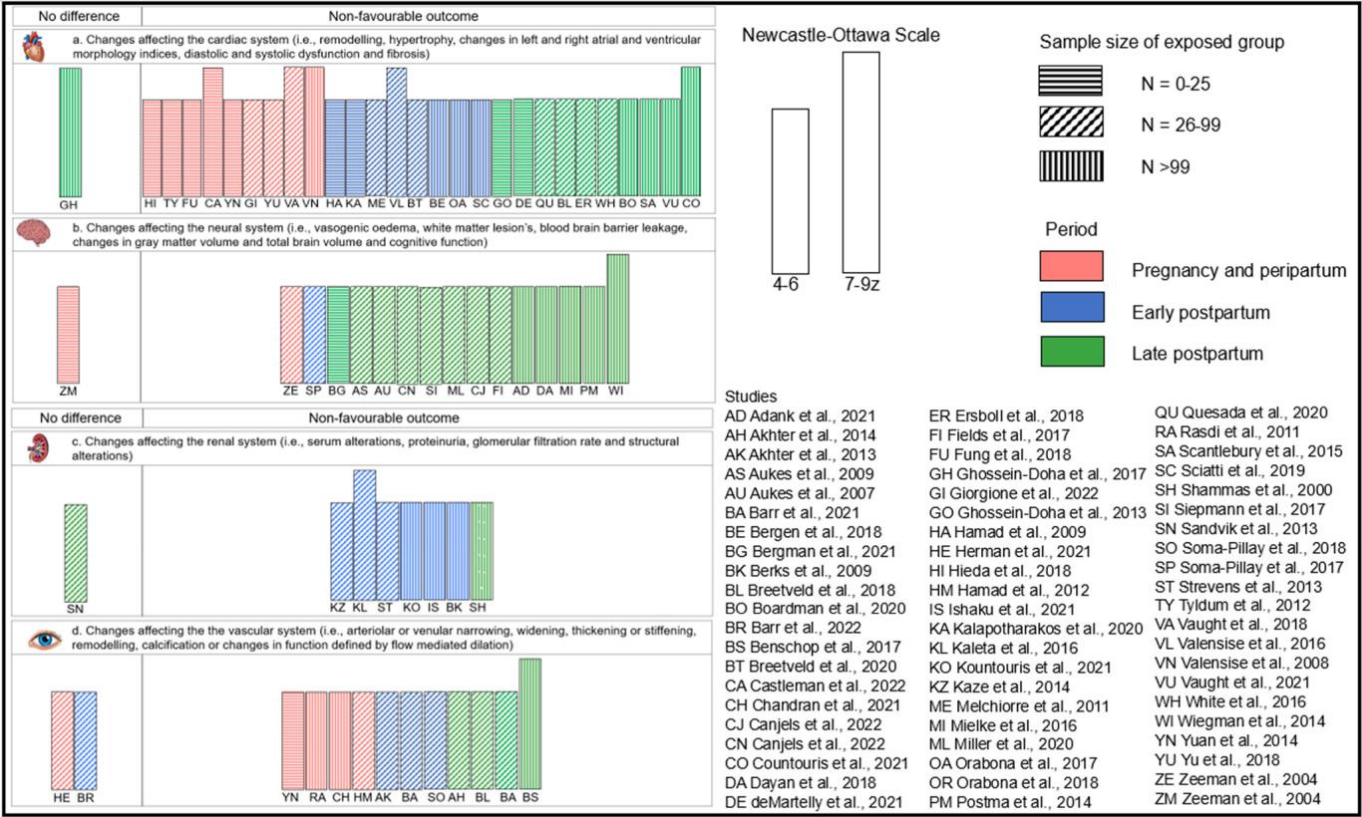
Harvest plot of studies based on vote-counting approach. Direction of effect was defined for each outcome domain and type of effect size measure was labelled as ‘non-favourable outcome’ or ‘no difference’. Results are separated by outcome domain and bars are coded by colour representing the timeframe of the study, pattern representing the sample size of exposed group, and height representing the risk of bias score. Only high and moderate risk of bias studies were included in the analysis. Authors are represented by initials. *N*, Number of participants.

Of the 76 studies, 12 reported blood pressure only, while 64 higher quality studies reported target organ changes (i.e. low to moderate risk of bias). Out of these, ‘non-favourable’ outcomes from hypertensive pregnancy were reported in 57 higher quality studies (90.5%) and ‘no differences’ were reported in seven studies (9.5%), *Figure 2*.

### Outcomes

#### Cardiac

**Figure 3:**
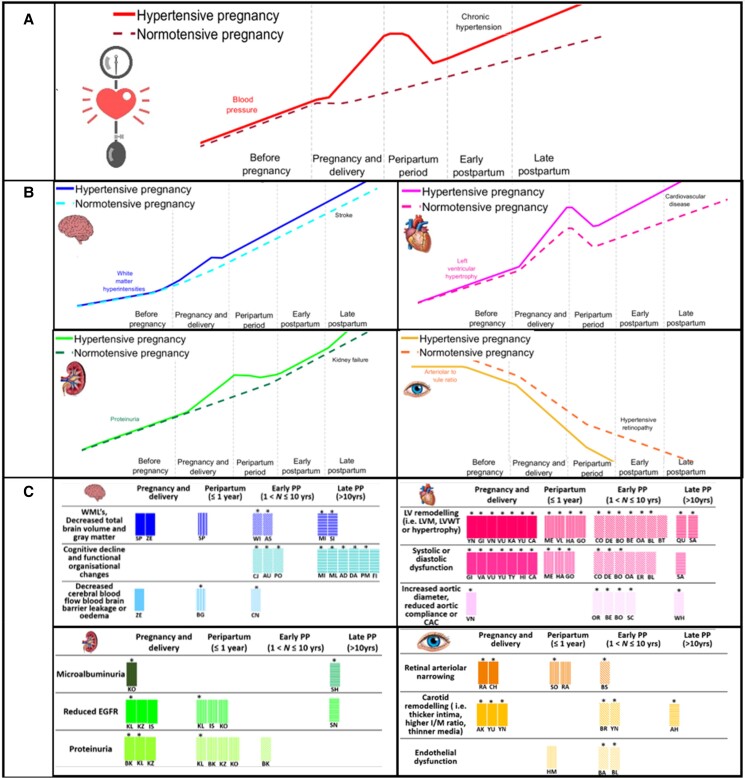
(*A*) A conceptual figure outlining theoretical blood pressure trajectories of across key periods during hypertensive and healthy pregnancies. The bold and dashed lines represent the predicted disease trajectories for women with hypertensive pregnancy and women with normotensive pregnancy, respectively. (*B*) Theoretical trajectories of target organ damage across hypertensive and healthy pregnancy based on the evidence provided in this review. (*C*) An evidence-based plot of the types of target damage caused by hypertensive pregnancies. Significant differences are represented by *. Different time periods are represented by different patterned bars. Papers corresponding to each result are represented by then initials of the first author.

Cardiac alterations were reported in 11 studies during pregnancy and early postpartum (39.2%) and 17 studies (60.7%) late postpartum. All studies in this section were categorized as low (17.8%) to moderate risk of bias (82.2%). Non-favourable outcomes of hypertensive pregnancy were reported in 27 higher quality studies^12–15,17,18–21,23–29,31,33–36,42,45,87^ and ‘no significant differences’ in 3 higher quality studies.^22,30,32^ During pregnancy and delivery, seven studies reported increased left ventricular remodelling in women with hypertensive pregnancies including increased left ventricular mass, hypertrophy, or wall thickness,^12,13,18,19,21,26,42^ seven studies reported systolic or diastolic dysfunction.^12–15,17,18,26^ Up to 1 year postpartum, four studies reported increase left ventricular remodelling,^20,23,24,87^ and three reported systolic and diastolic dysfunction.^20,24,87^ From 1 to 10 years postpartum, seven studies reported increased left ventricular remodelling,^25,28,29,31–34^ six reported systolic or diastolic dysfunction.^28,31–34,46^ More than 10 years postpartum, two studies reported increased left ventricular remodelling,^35,36^ one study reported systolic or diastolic dysfunction (*Figure 3*).^36^

#### Vascular and microvascular

Vascular alterations were reported in seven studies during pregnancy and early postpartum (53.8%), and six studies (46.2%) late postpartum. All except one study (7.7%) were categorized as low (38.4%) to moderate risk of bias (53.8%). Non-favourable outcomes were reported in 11 of the higher quality studies^18,37,38,41–44,46,48–50^ and ‘no significant differences’ in 2 higher quality studies.^40,47^ During pregnancy and delivery, two studies reported significant narrowing of arteriolar or venular retinal calibres^37,38^ and three reported carotid remodelling such as thickening of the carotid intima, higher intima to media ratio, and thinner media.^18,42,43^ One study reported changes in the structure or function of the aorta including increased aortic diameter or reduced aortic compliance.^19^ Up to 1 year postpartum, two studies reported narrowing of arteriolar or venular retinal calibres,^38,44^ and one study reported decreased endothelial dysfunction.^41^ Between 1 and 10 years postpartum, one study found persistent retinal arteriolar narrowing,^49^ one study reported that carotid artery remodelling persisted,^42^ and two studies reported that endothelial dysfunction was still present.^46,48^ Four reported changes in the structure or function of the aorta.^27,29,31,45^ More than 10 years postpartum, one study reported increased carotid remodelling,^50^ and one study reported increased coronary artery calcification scores.^51^

#### Renal

Renal alterations were reported in four studies during pregnancy and early postpartum (50%) and four studies (50%) late postpartum. All were categorized as low (50%) to moderate risk of bias (50%). Non-favourable outcomes were reported in seven higher quality studies^52–57,59^ and ‘no significant differences’ in one higher quality study.^58^ At pregnancy and delivery, one study reported increased microalbuminuria,^52^ three studies reported decreased glomerular filtration rate,^53–55^ and three reported increased proteinuria.^53–55^ Up to 1 year postpartum, three studies reported reduced glomerular filtration rate,^52,54,55^ and four studies reported proteinuria.^52,53,55,57^ Between 1 and 10 years postpartum, one study reported proteinuria^57^ and over 10 years postpartum, one study identified microalbuminuria,^59^ and one reduced glomerular filtration rate.^58^

#### Neurological

Neurological alterations were reported in 5 studies during pregnancy and early postpartum (29.4%) and 12 studies (70.5%) late postpartum. All but one (5.9%) were categorized as low (29.4%) to moderate risk of bias (64.7%). Non-favourable outcomes from hypertensive pregnancy were reported in 16 higher quality studies^60,62,64–77^ and ‘no significant differences’ in one higher quality study.^63^ During pregnancy and early postpartum, two studies reported structural changes in the brain including increased numbers of white matter lesions^62,64^ and one study reported changes affecting cerebral blood flow or the blood brain barrier.^62^ Up to 1 year postpartum, one study reported structural changes in the brain,^60^ and one reported blood brain barrier alterations^60^. Between 1 and 10 years postpartum, two studies reported structural neural changes,^67,68^ three studies reported cognitive or functional organization changes,^65,66,71^ and one study reported changes in cerebral blood flow or the blood brain barrier.^69^ More than 10 years postpartum, two studies reported structural changes in the brain,^70,77^ and six studies reported cognitive decline or functional organization changes.^72–77^

#### Comparison with blood pressure trajectories

Blood pressure trajectories were reported in five studies during pregnancy and early postpartum (50%), and five studies (50%) late postpartum.^7,16,78–86^ All studies in this section were categorized as low (60%) to moderate risk of bias (40%). These demonstrated high blood pressures during pregnancy that reduced significantly in the early post-partum period and then rose again late postpartum.

## Discussion

This review synthesizes evidence of target organ damage in women with hypertensive pregnancies to understand the extent of organ involvement during pregnancy and evidence for persistence of these changes in the early and late postpartum periods. Fifty-seven high quality studies were found and demonstrate involvement of cardiac, cerebral, renal, and vascular systems from pregnancy for up to 40 years postpartum. Studies reporting longitudinal blood pressure changes were also identified and show more significant variation in blood pressure level than reported for target organ changes.

The extent to which blood pressure variations during a hypertensive pregnancy are associated with target organ changes has recently started to be realized. Our review highlights a large body of accumulated literature reporting, in particular, cardiac changes, including diastolic dysfunction^14,15,17,18^ and left ventricular remodelling.^14,88^ The same differences in cardiovascular geometry and function continue to be reported in cohorts throughout the early and later post-partum periods^12,25,26,28^ suggesting that these changes are not completely reversed after resolution of the specific haemodynamic demands of pregnancy.

At 6 months, the cardiac effects of hypertensive pregnancy appear to be less pronounced than observed in pregnancy^21,42^ although significant differences in left ventricular and atrial dimensions, as well as diastolic function, are evident.^20^ At 5–10 years postpartum,^31,34,35^ several studies report increases in left ventricular mass index and higher left atrial volume related to a hypertensive pregnancy. At this time point, a distinct pattern of left ventricular mass distribution, identified with computational modelling, specifically related to a hypertensive pregnancy history has been reported, which is independent of blood pressure level.^31^ Women with a history of hypertensive pregnancy, who have become hypertensive in later life, also appear to have higher rates of left ventricular hypertrophy compared with those who are hypertensive but had normotensive pregnancies.^34,35^

Vascular changes relevant to hypertensive target organ damage are also consistently reported. Retinal arterio-venous ratio is lower in the first and second trimesters of pregnancy in hypertensive pregnancies,^37–39^ proposed to relate to local vasoconstrictive effects of inflammation and nitric oxide depletion. The usual macular thinning observed in pregnancy also does not appear to occur in those who develop hypertensive pregnancy, which may lead to a relative hypoperfused retinal environment.^40^ Our review highlights that these seem retinal changes remain present from 1- to 7-year postpartum.^44,49^ Other less clinically relevant microvascular markers have also been shown to be altered for years after pregnancy including endothelial function^17,41,47^ and microvascular structure.^31^

Macrovascular carotid changes, including intima media thickness and stiffness, do appear to improve slightly in the months postpartum, suggesting modifiability, but remain significantly different between normotensive and hypertensive populations^42,43^ for up to 10–11 years postpartum.^48,50^ New onset impairment in kidney function and proteinuria is another pathognomonic characteristic of pre-eclampsia and likely to have a vascular origin. This review highlights a continuing high prevalence but largely related to the early post-partum period.^57–59^ At 6 months, decreased endothelial glomerular filtration rate^54^ and proteinuria^53,57^ continues to be reported although, at 2 years postpartum, proteinuria is not evident^57^ with no significant difference in glomerular filtration rate at 10 years^58^ despite evidence of microalbuminuria.^59^

We found less reports related to cerebral and cerebrovascular outcomes but greater diversity in investigated measures. Pre-eclampsia is linked with an unusual high risk of seizures compared with hypertension at other times in life^89^ thought to relate to specific pregnancy-related changes in cerebrovascular autoregulation and blood–brain barrier function.^90^ Interestingly, blood barrier function also appears to vary outside of pregnancy with variation in leakage rate and fractional leakage volume in grey and white matter identified 5–8 years postpartum in women who had pre-eclampsia.^66^

Clinically relevant cerebrovascular and structural changes are also consistently reported postpartum. White matter lesions are evident during pregnancy in women with hypertension and remain present at a two-fold higher rate postpartum^67^ for at least 7 years.^68^ These are accompanied by alterations in white matter structure in particular in the temporal lobes,^70^ grey matter integrity, and total brain volume,^60,62,64,67,68,70,71,77^ with the degree of impairment increasing with time from pregnancy.^70^ However, cerebrovascular disease burden may be modifiable as one study reported prevalence of white matter lesions reducing from 61.7% at delivery to 47.9% at 1 year postpartum.^44^ As high intensity lesions on magnetic resonance imaging reflect protein and fluid extravasation, this change may relate to resorption and reversibility. Ability to improve or protect cerebral integrity is likely to be important as this review has identified increasing reports that women with hypertensive pregnancies display specific cognitive functional impairments. Increased emotion- and cognition-related symptoms and worse performance on cognitive tests for up to 10 years postpartum^69,71,74,77^ have been aligned with differences in functional connectivity in the limbic regions and the prefrontal cortex.^69^

Given the extent of target organ damage in women with hypertensive pregnancies, trialled interventions should be targeted to reverse damage and improve clinical outcomes in the peri-partum period. Recently, a large-scale randomized controlled trial, SNAP-HT (Self-Management of Postnatal Hypertension, NCT02333240) tested a short period of blood pressure self-management after hypertensive pregnancy.^91^ This trial resulted in lower blood pressure after 6 months which was maintained 3.6 years later.^91^ Therefore, by tightly controlling blood pressure in the peri-partum period, this trial had a long-term impact on risk of hypertension. Following this trial, POP-HT (Physician Optimised Post-partum—Hypertension Treatment) was developed to investigate this phenomenon further^92^ including whether some of the target organ changes, described in the current review, could be prevented or reversed. The results of this trial are yet to be released but demonstrate how knowledge of temporal patterns of target organ damage could be used for design of future trials at different timepoints postpartum.

### Limitations

This review has several limitations. Only studies published up to February 2023 were included and more recent studies or those in progress will not have been captured within the search strategy. Nevertheless, the systematic approach adopted will have ensured a very broad capture of all relevant studies published to date. Therefore, the findings can be regarded as the most comprehensive and up to date summary of publically available evidence related to hypertensive target organ changes during and after hypertensive pregnancy.

Due to the lack of quantitative outcomes there was no attempt to perform a meta-analysis to estimate effect sizes for different variables. This limits the conclusions that can be drawn but within the discussion we have undertaken a qualitative comparison of effect sizes at different time points for measures where sufficient evidence was available. Additionally, given the observational nature of studies in this review and the lack of consideration of potential confounding factors such as maternal age, lifestyle, and environment in many of the papers, a cause-and-effect relationship cannot be inferred from the findings presented here. Our review identifies a potential need for more detailed and comprehensive evaluation of end organ changes, taking into consideration potential confounding factors, during and after hypertensive disorders of pregnancy.

Furthermore, most studies only reported one type of outcome, and most did not use hypertensive control groups of women who had normotensive pregnancies. This can introduce selection bias based on the groups of women approached to participate and the requirement for specific tests, including imaging investigations, which may be applicable within only certain women. Therefore, any co-association of outcomes within the same individuals, and their interdependence with blood pressure variation, has not been explored except in a limited number of studies. No studies reported sufficient information on ethnicity to be able to draw any conclusions related to these factors or to determine the likely generalizability of the observations. Finally, this review is not registered with PROSPERO does limit the ability of the reader to assess for bias during the review process. Nonetheless, the authors of this article had no conflicts of interest and used a systematic approach to ensure the results are objective.

## Conclusions

This review highlights that women who have a hypertensive pregnancy already have evidence of target organ damage, typical for hypertensive disease, across the cardiac, vascular, renal, and cerebral systems during pregnancy. Although there is some partial resolution of these target organ changes postpartum, key differences persist, both during the early and late post-partum periods. This is despite significant variability in blood pressure with early normalization followed by a subsequent acceleration of hypertensive disease trajectories. The high prevalence of target organ damage peri-partum suggests future work understanding its role in disease and risk development will be of interest. Patterns of target organ changes peri-partum may identify women at highest risk of future disease and interventions to reverse these changes need to be trialled to understand whether clinical outcomes can be improved.

## Authors’ contributions

Review design and development was conducted by H.R.C., L.B., P.L., W.L., and A.J.L. Literature search, selection and data extraction was conducted by H.R.C., L.B., and P.D.S. Risk of bias assessment was completed by P.D.S and H.R.C. Data synthesis was completed by H.R.C. and P.D.S. The main body of the review was written by H.R.C., L.B., and P.L. Visualizations were generated by H.R.C., M.A., P.D.S, P.L., A.J.L., and W.L. Supervision was under P.L., W.L., and A.J.L. All authors critically appraised and revised the article. All authors approved the content of the final manuscript.

## Data Availability

Template data collection forms, data extracted from included studies, data used for all analyses, analytic code, any other materials used in the review are available upon reasonable request from the corresponding author.
